# Study of the Ternary Mixture of Methanol/Formamide/Acetonitrile via Solvatochromic Probes

**DOI:** 10.3390/molecules29010246

**Published:** 2024-01-02

**Authors:** Nelson Nunes, Ruben Elvas-Leitão, Filomena Martins

**Affiliations:** 1 Departamento de Engenharia Química, Instituto Superior de Engenharia de Lisboa, Instituto Politécnico de Lisboa, Rua Conselheiro Emídio Navarro, 1959-007 Lisboa, Portugal; ruben.leitao@isel.pt; 2 Centro de Química Estrutural, Institute of Molecular Sciences, Faculdade de Ciências, Universidade de Lisboa, Ed. C8, Campo Grande, 1749-016 Lisboa, Portugal; 3 Departamento de Química e Bioquímica, Faculdade de Ciências, Universidade de Lisboa, Ed. C8, Campo Grande, 1749-016 Lisboa, Portugal

**Keywords:** ternary mixture, methanol, formamide, acetonitrile, solvatochromic probes, Kamlet–Taft parameters, solvation

## Abstract

Following previous studies, the ternary mixture of methanol/formamide/acetonitrile (MeOH/Formamide/MeCN) was studied using the UV-Vis absorption spectra at 298.15 K with a set of five probes, 4-nitroaniline, 4-nitroanisole, 4-nitrophenol, *N*,*N*-dimethyl-4-nitroaniline and 2,6-diphenyl-4-(2,4,6-triphenyl-1-pyridinio)phenolate (Reichardt betaine dye), for a total of 22 mole ternary fractions. In addition, nine mole fractions of the underling binary mixtures, MeOH/Formamide and Formamide/MeCN were also tested. Spectroscopic results were used to model the preferential solvation order for each probe in the mixtures. The Kamlet–Taft solvatochromic solvent parameters, *α*, *β*, and π*, were also computed through the use of the solvatochromic shifts of the five probe indicators. Moreover, discrepancies in the spectroscopic behavior of 4-nitrophenol in formamide-rich mixtures were observed and analyzed.

## 1. Introduction

The use of solvent mixtures in various chemical applications has been a consistent practice for many years now. Their physicochemical characterization has also seen significant development, largely due to their critical role in several methods such as liquid chromatography. However, studies focusing on solvent mixtures beyond binary ones are much less common. One of the exceptions is the research involving solvatochromic probes, an area that could benefit enormously from further exploration.

Solvatochromism, which refers to the shift in a molecule’s absorption spectrum due to changes in the solvent environment, serves as an important tool for examining solute–solvent interactions. The complexity of solvent mixtures presents a challenge due to the increased intricacy of solvent–solvent interactions as the number of components rises. Despite this, solvatochromic probes can offer valuable insights into these interactions [1]. They can be used to identify different polarity parameters, such as the Kamlet–Taft solvatochromic parameters, and quantify preferential solvation phenomena.

**Figure 1:**
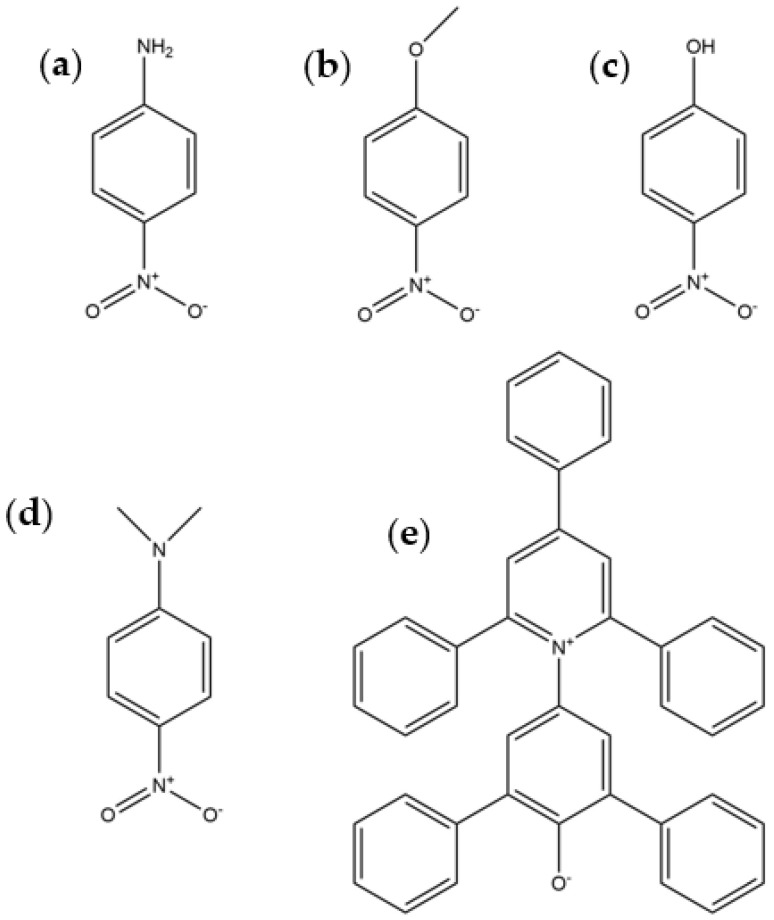
Used probes: (**a**) 4-nitroaniline; (**b**) 4-nitronisole; (**c**) 4-nitrophenol; (**d**) *N*,*N*-dimethyl-4-nitroaniline; (**e**) Reichardt’s betaine dye (2,6-diphenyl-4-(2,4,6-triphenyl-1-pyridinio)phenolate) betaine (30).

In this work, five solvatochromic probes, 4-nitroaniline, 4-nitroanisole, 4-nitrophenol, *N*,*N*-dimethyl-4-nitroaniline and Reichardt’s betaine dye (2,6-diphenyl-4-(2,4,6-triphenyl-1-pyridinio)phenolate), were used to study the ternary mixture of methanol/formamide/acetonitrile (MeOH/Form/MeCN) (Figure 1).

The selection of the mixture components is rooted in the versatility of three distinct organic solvents, commonly employed in both laboratory and industrial settings, each possessing unique properties. Specifically, methanol serves as a protic polar solvent, characterized by the presence of hydrogen-bonding to oxygen (-OH). Formamide, another protic polar solvent, distinguishes itself with hydrogen-bonding to nitrogen (-NH), while also featuring formamide as a polar aprotic solvent. Additionally, we have previously studied this mixture from a structural point of view, with the report of refractive indices and densities in the whole composition range [2]. Finally, the Kamlet–Taft parameters of one of the underling binary mixtures (methanol–acetonitrile) have also been successfully attained [3].

The initial step of the envisaged procedure involves computing the Kamlet–Taft solvatochromic parameters *α*, *β*, and π*. Comprehensive information regarding the calculation methodologies employed and the origins of these scales can be found in our earlier publications [3]. In essence, the π* scale measures the dipolarity/polarizability of the solvent, assessing all nonspecific interactions between the probe and the solvent [4]. This evaluation entails comparing the probe’s behavior in a specific solvent with its behavior in two reference solvents: cyclohexane, where π* equals to 0, and dimethylsulfoxide, where π* equals to 1. Since two probes (4-nitroanisole and *N*,*N*-dimethyl-4-nitroaniline) can be used for this purpose, two subscales can be generated (Equations (1) and (2)) [5].

(1) $$\pi_{OMe}^{*}=\frac{\left[\bar{\sigma }{\left(4-nitroanisole\right)}_{solvent}-34.12\right]}{-2.4}$$

(2) $$\pi_{NMe2}^{*}=\frac{\left[\bar{\sigma }{\left(N,N-dimetyl-4-nitroaniline\right)}_{solvent}-28.18\right]}{-3.52}$$

where $\bar{\sigma }$ is the wavenumber of the probe’s associated maximum energy transition band.

The *β* scale quantifies specific probe–solvent interactions associated with the hydrogen bond acceptor basicity of solvents [6,7]. This scale evaluates *β* for the examined solvent by comparison with two reference solvents: cyclohexane (with *β* = 0) and hexamethylphosphoramide (with *β* = 1). For this scale, two pair of probes can be used (4-nitroanisole/4-nitrophenol and *N*,*N*-dimethyl-4-nitroaniline/4-nitroaniline), generating two different subscales *β*_OH_ and *β*_NH2_ (Equations (3) and (4)).

(3) $$\beta_{OH}=\frac{\left[\left[1.043\bar{\sigma }{\left(4-nitroanisole\right)}_{solvent}-0.57\right]-\bar{\sigma }{\left(4-nitrophenol\right)}_{solvent}\right]}{2}$$

(4) $$\beta_{NH2}=\frac{\left[\left[0.9841\bar{\sigma }{\left(N,N-dimethyl-4-nitroaniline\right)}_{solvent}+3.49\right]-\bar{\sigma }{\left(4-nitroaniline\right)}_{solvent}\right]}{2.759}$$

Lastly, the α scale assesses specific probe–solvent interactions associated with the hydrogen bond donor acidity of solvents [8]. This evaluation is conducted by comparing probe–solvent interactions with probe–methanol interactions, where α equals 1. Two distinct subscales can be obtained, using betaine (30) and either 4-nitroanisole (for α_OMe_) or *N*,*N*-dimethyl-4-nitroaniline (for α_NMe2_) as hydrogen bond acceptor (HBA) probes (Equations (5) and (6)).

(5) $$\alpha_{OMe}=\frac{\left[\left[1.873\bar{\sigma }{\left(4-nitroanisole\right)}_{solvent}-74.58\right]+\bar{\sigma }{\left(betaine(30)\right)}_{solvent}\right]}{6.24}$$

(6) $$\alpha_{NMe2}=\frac{\left[\left[1.318\bar{\sigma }{\left(N,N-dimethyl-4-nitroaniline\right)}_{solvent}-47.7\right]+\bar{\sigma }{\left(betaine(30)\right)}_{solvent}\right]}{5.47}$$

The *α*, *β*, and π* values can be calculated from the average between each pair of subscales.

These parameters are an important tool in the study of solvents effects and their use is instrumental in the linear solvation energy relationships (LSERs) methodology. One example is the Kamlet–Taft equation/scale (Equation (7)) [9].

(7) $$\left(XYZ\right)={\left(XYZ\right)}_{0}+a\;\alpha +b\;\beta +s\;(\pi^{*}+d\;\delta )\;$$

where *α*, *β*, and π* are the Kamlet–Taft polarity parameters described previously, (*XYZ*) term is the result of a solvent-dependent chemical process, and (*XYZ*)_0_ is the value of the chemical process in a solvent medium without interactions. The parameter *δ* is a correction used in halogenated and for aromatic solvents and finally the coefficients a, b, s, d are solvent-independent coefficients that map the corresponding impact of the above-mentioned descriptors on the chemical process of interest (*XYZ*).

When discussing the behavior of solvatochromic probes within mixtures, it is acknowledged that preferential solvation phenomena may occur. Over time, numerous models have been proposed to explain and quantify these phenomena [10,11,12,13,14]. The Bosch and Rosés preferential solvation model stands out among these, having been successfully applied in the study of numerous binary mixtures [10,15,16,17,18,19,20,21,22]. This model is predicted on a two-step solvent exchange process, represented by the following Equilibria (8) and (9):

I(S1)*_m_* + *m* S2 ⇆ I(S2)*_m_ + m* S1 (8)

I(S1)*_m_* + (*m*/2) S2 ⇆ I(S12)*_m_ +* (*m*/2) S1 (9)

where ‘I’ denotes the solute or indicator, while ‘S1’ and ‘S2’ are the two pure solvents. ‘S12′ refers to a novel solvent entity or a “solvent complex”, which is the result of the interaction between solvents 1 and 2. The variable ‘*m*’ represents the number of solvent molecules participating in the exchange process within the solute’s cybotactic region, that is, within the solvation microsphere of the solvatochromic probe. The model only needs to account for the molecules within this region, as we have previously demonstrated [23]. ‘I(Si/j)’ denotes the indicator fully solvated by either solvent i or j, or both.

Each equilibrium constant can be related to a preferential solvation parameter, *f*. For Equation (8), the simplest situation of exchange of solvent molecules, *f*_2/1_ measures the propensity of the indicator (or probe) to be preferentially solvated by solvent S2 than by solvent S1 and represents the mole fraction distribution of the solvent between the solute’s cybotactic region (*x*^S^) and the bulk solvent (*x*^0^) (Equation (10)).

(10) $$f_{2/1}=\frac{\frac{x_{2}^{S}}{x_{1}^{S}}}{{\left(\frac{x_{2}^{0}}{x_{1}^{0}}\right)}^{m}}$$

In Equation (11), parameter *f*_12/1_ quantifies the solvating ability of the S12 complex relative to solvent S1. $x_{12}^{S}$ represents the mole fraction of the binary complex S12 within the solvation microsphere of the probe.

(11) $$f_{12/1}=\frac{\frac{x_{12}^{S}}{x_{1}^{S}}}{\sqrt{{\left(\frac{x_{2}^{0}}{x_{1}^{0}}\right)}^{m}}}$$

The occurrence of property values above or below the value of the solvatochromic properties for the pure components (provided that this variation surpasses experimental uncertainty) points out to the presence of significant solvent–solvent interactions and hence implies a value of *m* equal or greater than 2, therefore corresponding to a mixture with synergistic effect.

The extension of the Bosch and Rosés model to ternary mixtures has been presented and applied by authors in other studies [3,23]. The methodology resulted in an increase in the number of the equilibria involved and the complexity of the subsequent mathematical treatment. It required the simultaneous consideration of balances (8) and (9), (12), and (14), and (13) and (15), which correspond to the extensions to the ternary mixture of the general two-stage solvent exchange model.

I(S1)*_m_* + *m* S3 ⇆ I(S3)*_m_* + *m* S1 (12)

I(S1)*_m_* + *m*/2 S3 ⇆ I(S13)*_m_* + *m*/2 S1 (13)

I(S2)*_m_* + *m* S3 ⇆ I(S3)*_m_* + *m* S2 (14)

I(S2)*_m_*+ *m*/2 S3 ⇆ I(S23)*_m_* + *m*/2 S2 (15)

Additionally, it is also possible to conceive the formation of a ternary solvating complex with the solute I(S123), which can be represented by the following equilibria (16), (17) and (18):

I(S1)*_m_* + *m*/3 S2 + *m*/3 S3 ⇆ I(S123)*_m_* + 2*m*/3 S1 (16)

I(S2)*_m_* + *m*/3 S1 + *m*/3 S3 ⇆ I(S123)*_m_* + 2*m*/3 S2 (17)

I(S3)*_m_* + *m*/3 S1 + *m*/3 S2 ⇆ I(S123)*_m_*+ 2*m*/3 S3 (18)

For each of these equilibria, as in the Bosch and Rosés original model, it is possible to define an equilibrium constant that relates the solvent’s mole fraction in the solvation microsphere of the probe with its mole fraction in the bulk. These equilibrium constants can be once more associated with preferential solvation parameters, *f*.

In the case of the formation of the ternary solvent complex I(S123), its preferential solvation relative to solvent S1 can be obtained from Equation (19).

(19) $$f_{123/1}=\frac{\frac{x_{123}^{S}}{x_{1}^{S}}}{\frac{\sqrt[{3}]{{\left(x_{2}^{0}x_{3}^{0}\right)}^{m}}}{\sqrt[{3}]{{\left(x_{1}^{0}\right)}^{2m}}}}$$

where $x_{123}^{S}$ stands for the mole fraction of the ternary complex S123.

A solvatochromic property (*Y*) in a mixture can be expressed as the sum of the contributions of each solvent entity in the solute´s cybotactic region, which is given by the product of the corresponding mole fractions by the property value for each entity, *Y*i (Equation (20)):

(20) $$Y=Y_{1}x_{1}^{S}+Y_{2}x_{2}^{S}+Y_{3}x_{3}^{S}+Y_{12}x_{12}^{S}+Y_{13}x_{13}^{S}+Y_{23}x_{23}^{S}+Y_{123}x_{123}^{S}$$

Mole fractions in the cybotactic region must next be converted into known variables based on the preferential solvation parameters, *f*, previously defined, given that the sum of all mole fractions in the cybotactic region and in the solvent’s bulk has to be equal to one. After the necessary simplifications, one obtains the preferential solvation expression as Equation (21):

(21) $$Y=\frac{Y_{1}{\left(x_{1}^{0}\right)}^{m}+Y_{2}\;f_{2/1}{\left(x_{2}^{0}\right)}^{m}+Y_{3}\;f_{3/1}{\left(x_{3}^{0}\right)}^{m}+Y_{12}\;f_{12/1}{\left(x_{1}^{0}x_{2}^{0}\right)}^{\frac{m}{2}}+Y_{13}\;f_{13/1}{\left(x_{1}^{0}x_{3}^{0}\right)}^{\frac{m}{2}}+Y_{23}\;f_{23/1}{\left(x_{2}^{0}x_{3}^{0}\right)}^{\frac{m}{2}}+Y_{123}\;f_{123/1}{\left(x_{1}^{0}x_{2}^{0}x_{3}^{0}\right)}^{\frac{m}{3}}}{{\left(x_{1}^{0}\right)}^{m}+f_{2/1}{\left(x_{2}^{0}\right)}^{m}+f_{3/1}{\left(x_{3}^{0}\right)}^{m}+f_{12/1}{\left(x_{1}^{0}x_{2}^{0}\right)}^{\frac{m}{2}}+f_{13/1}{\left(x_{1}^{0}x_{3}^{0}\right)}^{\frac{m}{2}}+f_{23/1}{\left(x_{2}^{0}x_{3}^{0}\right)}^{\frac{m}{2}}+f_{123/1}{\left(x_{1}^{0}x_{2}^{0}x_{3}^{0}\right)}^{\frac{m}{3}}}$$

Whenever *f*_i/j_ is close to 1, an ideal mixture is present, and one can consider that there is no preferential solvation and thus no synergism. If *f*_i/j_ > 1, this implies that the probe is preferentially solvated by solvent i rather than by solvent j. On the other hand, if *f*_i/j_ < 1, the probe is better solvated by solvent j.

Finally, since all parameters are associated with the same solvent, S1, it is possible to establish preferential solvation scales in terms of the measured solvatochromic property, *Y*.

## 2. Results and Discussion

### 2.1. Preferential Solvation Analysis

Data related to the experimental wavenumbers of each probe’s absorption band in the different mixtures are available in Supplementary Material (SI) Tables S1–S4. Data for the binary mixture methanol/acetonitrile were taken from prior studies [3,23,24] and were also presented. The Bosch and Rosés preferential solvation model parameters obtained through nonlinear iterative fitting of each probe’s results in the binary mixtures can be found in SI Tables S5–S9. Table S10 refers to the ternary system and includes the adjusted parameters using our modified version of the Bosch and Rosés preferential solvation model [23]. In this case, the initial *Y* values were those obtained from the binary adjustments. The values highlighted in bold in Tables S5–S10 are the outcomes of mathematical manipulations of other parameter values.

**Figure 2:**
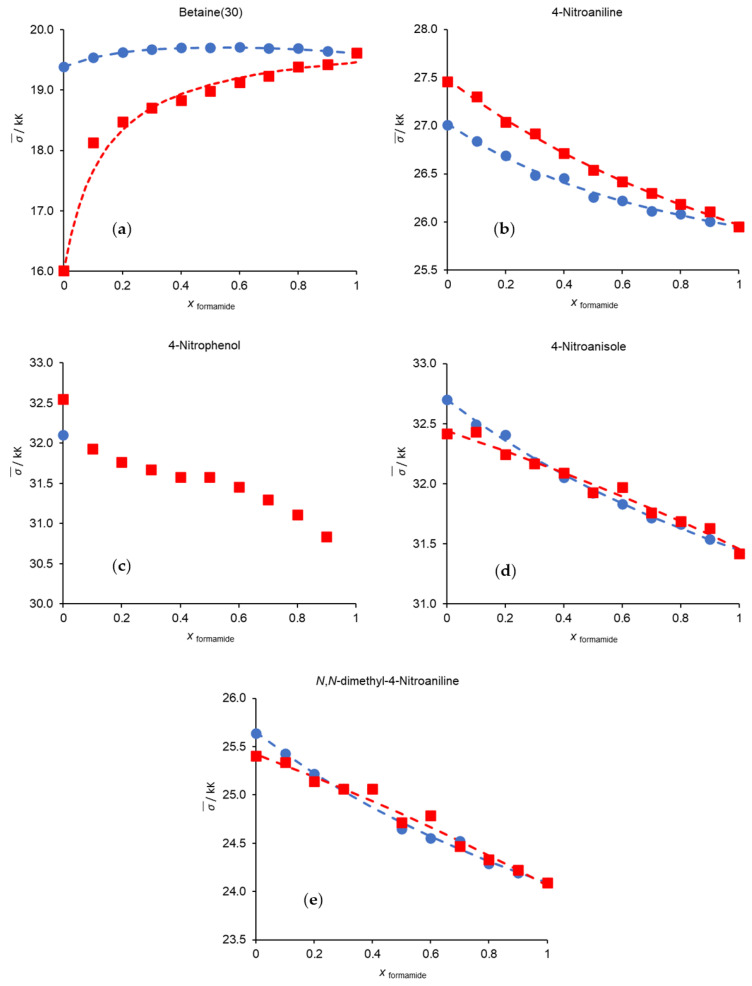
Variation in wavenumbers of each probe with the mole fraction of formamide at 298.15 K. (**a**) Betaine (30); (**b**) 4-nitroaniline; (**c**) 4-nitrophenol; (**d**) 4-nitronisole; (**e**) *N*,*N*-dimethyl-4-nitroaniline: (●) methanol/formamide; (■) formamide/acetonitrile. The dashed lines represent the best fits resulting from the application of the Bosch and Rosés preferential solvation model. The values for methanol/acetonitrile can be found in our previous works [3,23,24].

Figure 2 illustrates the changes in the experimental wavenumbers of each probe in the studied binary solvent mixtures. The dashed lines represent the optimal fitting functions calculated by the Bosch and Rosés model.

A quick overall analysis of the changes in the wavenumbers of the probes with solvent composition, as depicted in Tables S1 and S2 and Figure 2, shows that the wavenumber values of betaine (30) in the methanol/formamide mixture is higher than those of the pure components; in 4-nitrophenol, it was not possible to obtain values for pure formamide or any experimental point for the methanol/formamide mixture beyond pure methanol. This topic will be discussed later on.

**Figure 3:**
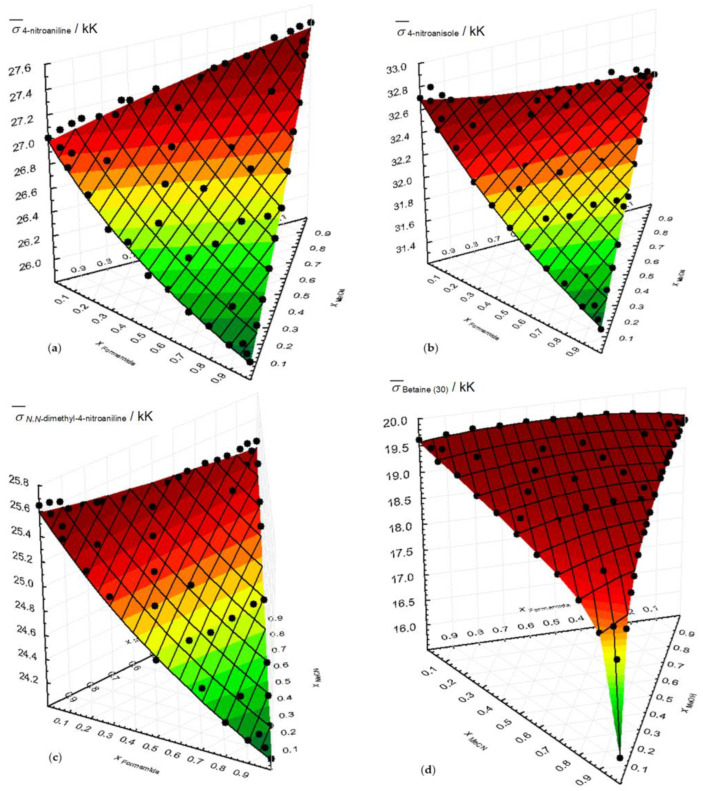
Variation in the wavenumbers of each probe with the mole fraction of the ternary mixture MeOH/Formamide/MeCN at 298.15 K. (**a**) 4-nitroaniline; (**b**) 4-nitronisole; (**c**) *N*,*N*-dimethyl-4-nitroaniline; (**d**) betaine (30). The surfaces result from the application of the ternary preferential solvation model.

Table S4 presents the variation in wavenumbers of each probe for the ternary of methanol/formamide/acetonitrile mixture. The same information is included in Figure 3, where, in addition to the ternary mixtures, the fractions of the binary mixtures are also shown.

In Figure 3, the good fit between the experimental results and the ternary surfaces generated by the parameters of the ternary solvation model is evident and will be discussed further. The lack of representation for the 4-nitrophenol probe is due to the limited availability of experimental points.

For a better understanding of the behavior of each individual probe in the studied binary mixtures, the Bosch and Rosés solvation model parameters (Tables S5–S9) must be carefully analyzed.

4-nitroaniline is clearly solvated by formamide (Table S6), which may indicate a strong specific interaction between the -NH_2_ groups of solvent and probe. In the case of 4-nitroanisole (Table S8), the probe is primarily solvated by formamide in the MeOH/Formamide mixture. However, in the Formamide/MeCN system, the mixture’s behavior, and the preferential solvation parameter’s value, along with its associated standard deviation, seem to indicate that this mixture is approaching ideal behavior.

Formamide and acetonitrile are the solvents that best solvate *N*,*N*-dimethyl-4-nitroaniline (Table S9), since both show a better capacity to solvate a probe with reduced capacity to establish specific interactions.

As for the betaine (30), and as stated before, there is a synergistic behavior in the MeOH/Formamide mixture. This is because formamide (HCONH_2_) interacts with the -OH groups of methanol to form complexes. These complexes exhibit greater polarity than the solvent components that gave rise to them which, considering the polarity characteristics of the ground and excited states of betaine, translates into a hypsochromic displacement of the betaine absorption band, i.e., a higher transition energy, and therefore a higher wavenumber than expected based on an ideal behavior.

The same type of analysis can be carried out for the ternary mixture using the ternary solvation model. The data for that are presented in Table S10 and summarized in Table 1.

In 4-nitroanline, the preferential solvation order predicted by the ternary model is different from the one based on the “binary” model. The attempt to explain the difference between the solvation order predicted by the “binary” (MeOH > MeCN) and the “ternary” (MeCN > MeOH) models for this mixture can be based on two different approaches: from a physicochemical point of view, it is possible to speculate that the presence of the third component (formamide), by interacting in a specific way with the other two components and more with MeOH than with MeCN may be responsible for the reversal of the solvation order, since, due to the strong interaction with formamide, methanol is less available to solvate the probe; from a mathematical point of view, this inversion of order can be explained by the fact that in binary mixtures there is some dispersion of the probes wavenumber and therefore a less well-defined order of solvation, an aspect that is solved by the increase in mole fractions resulting from the inclusion of the ternary mole fractions.

For 4-nitroanisole, the solvation order is the same, showing that the type of interactions present in the binary mole fractions is the same as that in the ternary mole fractions.

In *N*,*N*-dimethyl-4-nitroaniline, there is a slight change between the parameters of the binary model and the ternary model, allowing the ternary fractions for the reassessment of the relative position of the MeOH/MeCN complex and the establishment of the following sequence: Formamide ≈ MeCN > MeOH-MeCN complex > MeOH.

Finally, betaine (30) shows a behavior in ternary mole fractions that can only be explained by admitting an interaction between the three components of the mixture and the probe. Although the associated uncertainties are high, the *f*_123/1_ parameter has still statistical significance.

Despite the lack of synergism in the ternary mixtures for betaine (30) in any of the ternary mole fractions, the description of the experimental values is only possible if Equation (20) considers the ternary influence decoded by *Y*_123_ and *f*_123/1_, which corresponds to the presence of a ternary complex. It is interesting to evaluate why betaine (30) is the only probe to present ternary contributions with statistical significance and therefore with influence on the final value of the measured wavenumbers. This situation can possibly be explained by the fact that betaine (30), being a larger molecule with greater charge separation, may be more sensitive to a wider range of interactions with these solvents and mainly because this probe presents a greater variability in the measured property than that of the other probes tested. Thus, the difficulty may reside not on the other probes’ lack of sensitivity to a possible ternary contribution but on the fact that this effect may be negligible.

For all the probes, the ternary model reproduces the probes’ wavenumbers in each of the pure solvents.

### 2.2. Solvatochromic Parameters’ Analysis

In this section, the Kamlet and Taft parameters will be analyzed. The values of each parameter obtained from Equations (1)–(6) are presented in Tables S11–S14 of the Supplementary Material. Whenever possible, we chose to represent the parameters average values, except for *β* in the binary mixtures for reasons discussed below.

**Figure 4:**
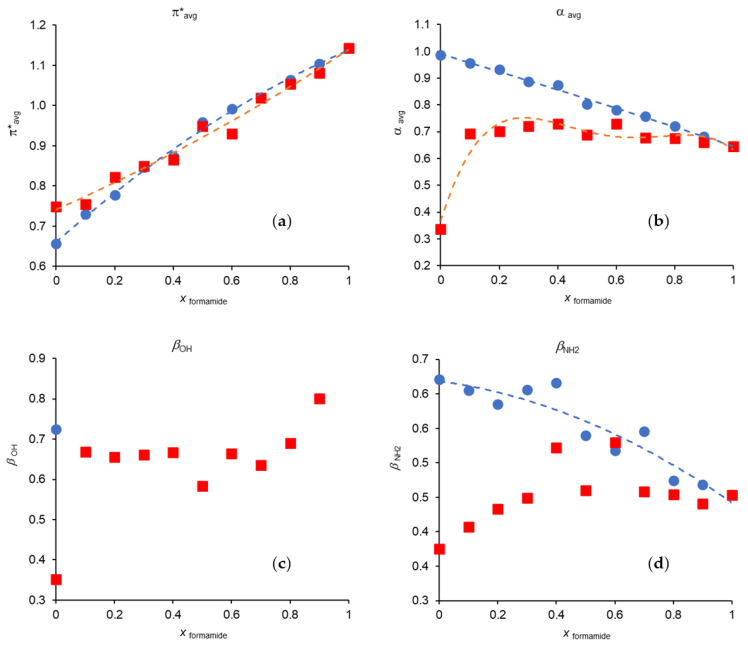
Variation of the Kamlet–Taft parameters with formamide mole fraction at 298.15 K: (**a**) π*_avg_; (**b**) α_avg_; (**c**) *β*_OH_; (**d**) *β*_NH2_; (●) methanol/formamide; (■) formamide/acetonitrile. The dashed lines represent the best fits resulting from the application of a polynomial adjustment. The values for methanol/acetonitrile can be found in our previous works [3,23,24].

Figure 4 represents the variation of π*_avg_, *α*_avg_, *β*_OH_ and *β*_NH2_ in each of binary mixtures studied in this work (data from Tables S11 and S12). The dashed lines represent empirical polynomial fitting functions. However, they are not used in *β*_OH_ for the mixture MeOH/Formamide due to the lack of points. They are also not present for Formamide/Acetonitrile in both *β*_OH_ and *β*_NH2_ due to the high dispersion of the data points.

The analysis of Figure 4 shows that π*_avg_ has a positive deviation from ideality in the mixture MeOH/Formamide and a negative deviation in Formamide/MeCN.

For α_avg_, there is a clear positive deviation in the mixture Formamide/MeCN with the maximum of the deviation being observed at mole fractions between 0.7 and 0.8 in MeCN. On the other hand, this parameter in the mixture MeOH/Formamide shows a behavior attributable to an ideal mixture.

As referred before, we did not use the average *β* values, mainly for two reasons: first, because the two scales are not correlated which seems to indicate that they are reading different interaction aspects; secondly, and due to the spectroscopic divergences of 4-nitrophenol, because there is a deficit of data points for the scale which uses this probe (*β*_OH_).

*β*_NH2_ values in Formamide/MeCN and MeOH/Formamide present positive deviations from ideality, although they exhibit a high dispersion. The increase in the basic character is also noticeable by the addition of a small amount of formamide to acetonitrile when the scale is based on the 4-nitrophenol/4-nitroanisole probes (*β*_OH_ scale), which does not occur in the same proportion when the scale refers to the 4-nitroaniline/*N*,*N*-dimethyl-4-nitroaniline probes *(β*_NH2_ scale). The reason for the increase has to do with the presence of the amide group of formamide.

**Figure 5:**
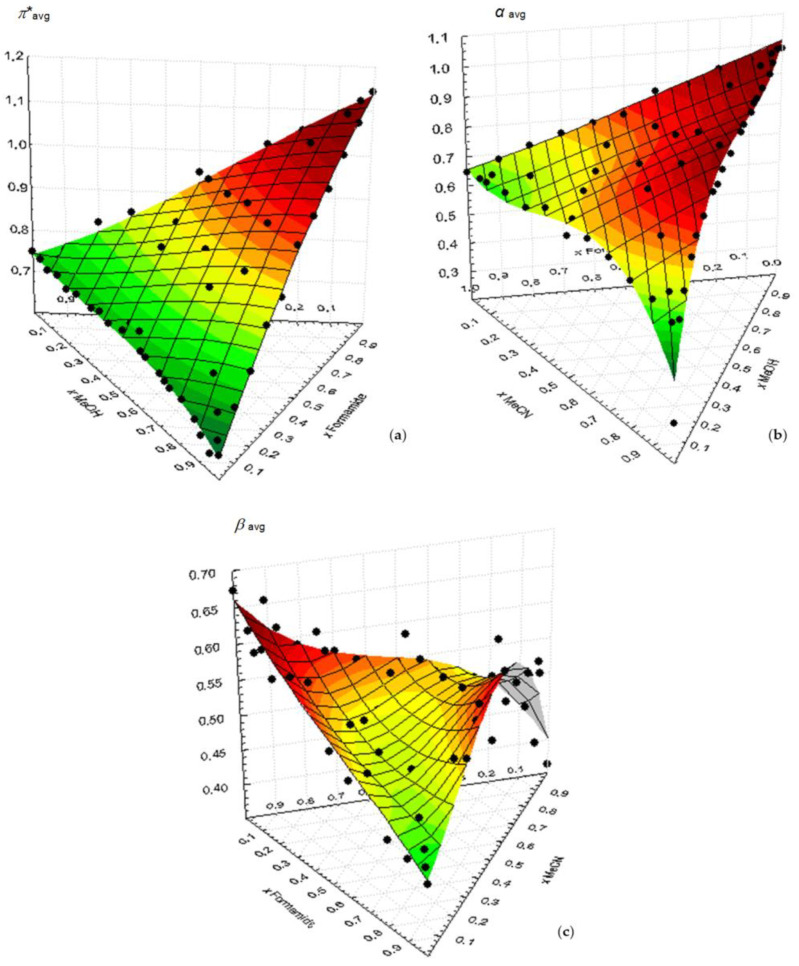
Variation in the wavenumbers of each Kamlet–Taft parameter with the mole fraction of the ternary mixture MeOH/formamide/MeCN at 298.15 K: (**a**) π*_avg_; (**b**) α_avg_; (**c**) *β*_avg_. The surfaces result from the application of a polynomial fitting function.

Figure 5 shows the variation in the wavenumbers of each Kamlet–Taft parameter with the mole fraction of the ternary mixture MeOH/Formamide/MeCN. The most important fact of the analysis of these plots is that their behavior can be mostly explained by the binary contributions, the “ternary component” also being clearly residual here and lying within the experimental uncertainty associated in each zone to the respective binary mixture.

A general interpretation of the results evidences the role of the specific solute−solvent hydrogen bonding interaction in the case of nonpolar−polar protic solvent mixtures, a fact also noted by other authors [25].

### 2.3. Analysis of the Spectroscopic Divergences of 4-Nitrophenol

One of the problems that emerged in the analysis of the 4-nitrophenol probe, particularly in fractions with a high percentage of formamide (see Tables S15–S17), was the appearance of a second band at wavelengths in the region 391–416 nm, which for some mole fractions was the only observable band. An initial evaluation of this band showed that, similarly to the characteristic band of 4-nitrophenol (λ ≈ 307–324 nm), the wavelength of this band also varied according to the solvent.

This problem has not been explored in the literature with reference to the value of the *β* parameter for formamide, which is a value established in bibliographic sources [26]. However, one of the original sources for the *β* parameter for formamide specifies that the value was estimated based on analogous solvents with similar groups [27].

The experimental behavior indicates that the formamide is somehow interacting with the probe, since in none of the mixtures without formamide this second band occurs. Our hypothesis is that formamide may have compromised the donor ability of the –OH group of 4-nitrophenol. To understand the nature of the interaction between the chemical entity possibly responsible for the appearance of this second band and the solvent, a correlation analysis was established between the wavenumbers of the second band and the solvatochromic parameters previously determined for the various mixtures, i.e., π*, *β*_NH2_ and α, for which this band was observed, adding up to 37 mole fractions (Equation (22)):

*σ* (kK) = 19.5 (±0.7) + 0.9 (±0.4) π* + 5.9 (±0.6) α − 0.5 (±0.5) *β*_NH2_

(100%) (98.5%) (100%) (70.0%)(22)

*R*^2^ = 0.959; *R*^2^_adj._ = 0.955; *s*_adj._ = 0.105; *F* = 255; *N* = 37

*R*^2^ is the determination coefficient, *R*^2^_adj._ is the (adjusted) determination coefficient which considers the degrees of freedom (number of experimental points and number of regression parameters), *s*_adj._ represents the (adjusted) standard deviation of the fit, *F* the Fisher–Snedecor parameter, and *N* is the number of points of the regression.

Disregarding the parameter with the least statistical significance (*β*_NH2_), the previous relationship takes the form of Equation (23):

*σ* (kK) = 19.4 (±0.9) + 0.9 (±0.6) π* + 5.6 (±0.6) α

(100%) (87.5%) (100%)(23)

*R*^2^ = 0.929; *R*^2^_adj._ = 0.914; *s*_adj._ = 0.124; *F* = 65; *N* = 37

However, this relationship presents a high correlation between parameters π* and α (*R*^2^ = 0.851). Thus, a new relationship was developed, using only 13 mole fractions, selected to decrease the intercorrelation between π* and α (Equation (24)):

*σ* (kK) = 20.7 (±0.4) + 5.0 (±0.5) α

(100%) (100%)(24)

*R*^2^ = 0.909; *R*^2^_adj._ = 0.900; *s*_adj._ = 0.133; *F* = 109; *N* = 13

The disappearance of π* in Equation (23) resulted from its low statistical significance. These three linear relationships (where the coefficients of the retained parameters are equivalent, which indicates a good robustness of the model) unequivocally show that there is a relationship between the wavenumber of the second band and α. This means that 4-nitrophenol in the presence of formamide may have a band that is sensitive to the acidity of the solvent.

**Figure 6:**
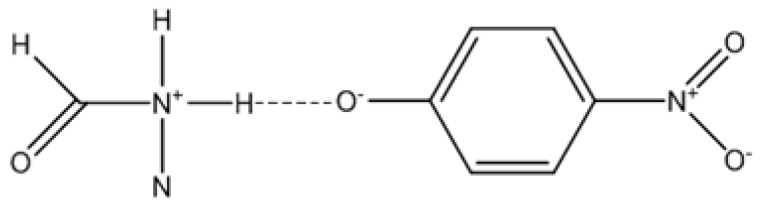
Formamide-4-nitrophenol interaction with proton transfer.

In fact, there are several published studies that support the existence of interactions between phenolic compounds and amides, as well as the formation of complexes. Studies using spectroscopic techniques [28], measurements of dielectric constants [29], and computational calculations [30] prove the stability of these complexes. However, the in-depth discussion about the nature of these interactions requires a combination of several techniques and subject to some debate to figure out whether the interaction involves a total proton transfer reaction [31] (vd. Figure 6).

**Figure 7:**
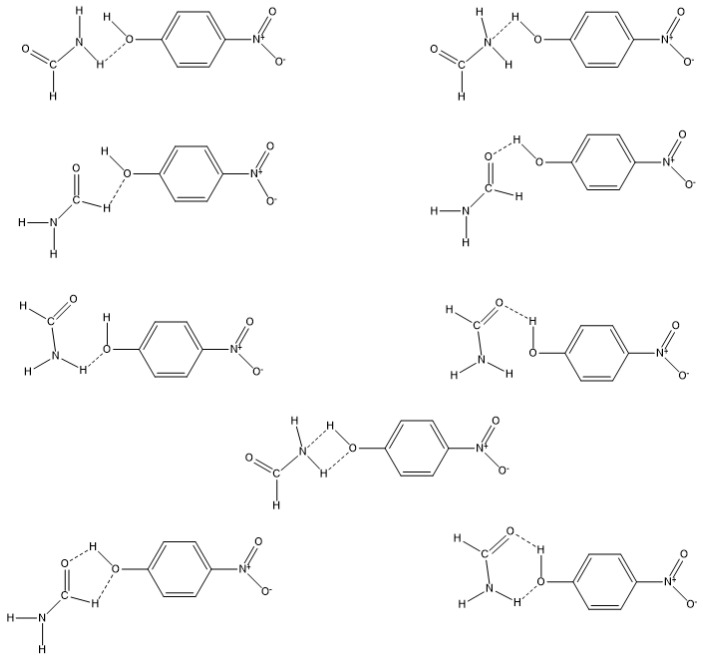
Schematic representation of potential formamide-4-nitrophenol interactions.

On the contrary, a formamide group (be it the -NH2 group, the –CH=O group, or both) preferentially interacts with the -OH group of the probe. These interactions may occur in linear intermolecular hydrogens bonds (more plausible) or by the formation of four-, five- and six-membered structures (less probable), as depicted in Figure 7.

It has been experimentally described by other authors that the solvent and the concentrations of amide and phenol influence the spectroscopic data in pure solvents [31]. Hence, and based on the set of results obtained from the analyzed mixtures, it is likely that at least a partial transfer of the proton from the probe to the solvent occurs, as suggested in Figure 6. This probe would therefore have a structure closer to Reichardt’s betaine (30), with a phenolate oxygen (a strong acceptor of hydrogen bonds) which would result in a greater susceptibility to the acidity of the solvent, as evidenced by the correlation analyses.

## 3. Materials and Methods

The experimental procedures were outlined in previous papers [3,23,24]. To summarize, solvent mixtures were prepared by weighing the components using a digital balance (accuracy of ±1 × 10^−4^ g). The solvents and solvatochromic probes listed in Table 2 were used as received. UV–Vis spectra were recorded at 298.15 K ± 0.1 K, using a double beam Thermo Nicolet Evolution 300 spectrophotometer; a representative spectrum of a probe in the ternary mixtures can be found in Figure S1 of the Supplementary Material. Th wavenumbers reported for each probe–solvent system represent the average of at least three independent measurements, with a standard deviation consistently below 0.07. Th solvatochromic parameters were calculated from the measured wavenumbers of each probe and expressed in kiloKaiser (1 kK = 10^3^ cm^−1^).

Nonlinear fitting to Equation (21) was performed using the Microsoft Excel^®^ add-in Solver. SYSTAT Software Inc., TableCurve 2D v5.01, and TableCurve 3D v.4.0 were employed for the computation of the model’s parameters and their associated uncertainties.

## 4. Conclusions

The ternary model applied in this work proved to be robust, accurately explaining the behavior of the ternary mole fractions and the correspondent solvation order. Another important finding is that the ternary model almost entirely reproduces the results of the Bosch and Rosés preferential solvation model for binary mixtures. However, a better accuracy was achieved when the probe had a high variation in its wavenumber, like in the case of betaine (30).

Applying the ternary model to the wavenumbers of the Reichardt probe, the formation of a “complex” involving an interaction between the three solvents in the ternary mixture was predicted. This seems to confirm the results based on molar volumes that also predicted an interaction of this type.

The solvatochromic parameters, π*, *α* and *β,* in ternary mixtures essentially result from the binary contributions.

The mixtures with formamide were the ones that showed the highest dispersion in the solvatochromic parameters, which were particularly evident in *β*.

Formamide and 4-nitrophenol can establish a strong interaction indicating that 4-nitrophenol can donate its hydroxylic proton to formamide, thereby forming a species sensitive to the acidity of the solvent.

## Figures and Tables

**Table 1:** Preferential solvation orders for all the studied probes using the ternary solvation model.

Probe	Order
4-Nitroaniline	Formamide > MeCN > MeOH
*N*,*N*-dimethyl-4-nitroaniline	Formamide ≈ MeCN > MeOH-MeCN complex > MeOH
4-Nitrophenol	---
4-Nitroanisole	MeCN ≈ Formamide > MeOH
Betaine (30)	MeOH-MeCN complex ≈ MeOH-Formamide complex > MeOH-Formamide-MeCN complex > Formamide > MeOH > MeCN

**Table 2:** Supplier and purity of solvents and probes used in the spectroscopic measurements.

Solvent/Probe	Supplier	Purity (%)
methanol	Riedel de Häen	>99.9
acetonitrile	Aldrich	>99.5
formamide	Aldrich	>99.5
4-nitroaniline	Aldrich	>99
4-nitroanisole	TCI	>98
4-nitrophenol	Merck	>99.5
*N*,*N*-dimethyl-4-nitroaniline	TCI	>98
2,6-diphenyl-4-(2,4,6-triphenylpyridinio)-1-phenolate (Reichardt’s betaine dye)	Aldrich	90

## Data Availability

Data are contained within the article.

## Supplementary Materials

The following supporting information can be downloaded at https://www.mdpi.com/article/10.3390/molecules29010246/s1, Table S1: Wavenumbers for each probe in methanol/formamide at 298.15 K (values in kK, 1 kK = 10^3^ cm^−1^). Table S2: Wavenumbers for each probe in formamide/acetonitrile at 298.15 K (values in kK, 1 kK = 10^3^ cm^−1^). Table S3: Wavenumbers for each probe in methanol/acetonitrile at 298.15 K. Data were taken from our previous work (values in kK, 1 kK = 10^3^ cm^−1^). Table S4: Wavenumbers for each probe in the ternary mixture methanol/formamide/acetonitrile at 298.15 K (values in kK, 1 kK = 10^3^ cm^−1^). Table S5: Adjusted parameters for the Bosch and Rosés model for betaine (30) at 298.15 K. Data referring to methanol/acetonitrile were taken from our previous work. Table S6: Adjusted parameters for the Bosch and Rosés model for 4-nitroaniline at 298.15 K. Data referring to methanol/acetonitrile were taken from our previous work. Table S7: Adjusted parameters for the Bosch and Rosés model for 4-nitrophenol at 298.15 K. Data referring to methanol/acetonitrile were taken from our previous work. Table S8: Adjusted parameters for the Bosch and Rosés model for 4-nitroanisole at 298.15 K. Data referring to methanol/acetonitrile were taken from our previous work. Table S9: Adjusted parameters for the Bosch and Rosés model for *N*,*N*-dimethyl-4-nitroaniline at 298.15 K. Data referring to methanol/acetonitrile were taken from our previous work. Table S10: Adjusted parameters for the Bosch and Rosés model for each probe in the ternary mixture methanol/formamide/acetonitrile at 298.15 K. Table S11: Kamlet–Taft parameters for the methanol/formamide mixture at 298.15 K. Table S12: Kamlet–Taft parameters for the formamide/acetonitrile mixture at 298.15 K. Table S13: Kamlet–Taft parameters for the methanol/acetonitrile mixture at 298.15 K. Data for this mixture were taken from our previous work. Table S14: Kamlet–Taft parameters for the methanol/formamide/acetonitrile mixture at 298.15 K. Table S15: Wavenumbers for the second band of 4-nitrophenol in the binary mixture methanol/formamide at 298.15 K (values in kK, 1 kK = 10^3^ cm^−1^). Table S16: Wavenumbers for the second band of 4-nitrophenol in the binary mixture formamide/acetonitrile at 298.15 K (values in kK, 1 kK = 10^3^ cm^−1^). Table S17: Wavenumbers for the second band of 4-nitrophenol in the ternary mixture methanol/formamide/acetonitrile at 298.15 K (values in kK, 1 kK = 10^3^ cm^−1^). Figure S1: Spectrum of betaine (30) in the ternary mixture methanol/formamide/acetonitrile (0.333/0.333/0.333).
