# Supplementary material for: Study of the Ternary Mixture of Methanol/Formamide/Acetonitrile via Solvatochromic Probes
Source: Molecules. 2024 Jan 2;29(1):246. doi: 10.3390/molecules29010246 (PMC10780662; doi:10.3390/molecules29010246)
Supplement: Supplementary file 1 [file molecules-29-00246-s001.zip › molecules-2769415-supplementary.pdf]

Supplementary Material

Study of the Ternary Mixture of Methanol/Formamide/Acetonitrile via Solvatochromic Probes

Nelson Nunes [1,2,*], Ruben Elvas-Leitão [1,2] and Filomena Martins [2,3,*]

[1] Departamento de Engenharia Química, Instituto Superior de Engenharia de Lisboa, Instituto Politécnico de Lisboa, Rua Conselheiro Emídio Navarro, 1959-007 Lisboa, Portugal; ruben.leitao@isel.pt

[2] Centro de Química Estrutural, Institute of Molecular Sciences, Faculdade de Ciências, Universidade de Lisboa, Ed. C8, Campo Grande, 1749-016 Lisboa, Portugal

[3] Departamento de Química e Bioquímica, Faculdade de Ciências, Universidade de Lisboa, Ed. C8, Campo Grande, 1749-016 Lisboa, Portugal

* Correspondence: nelson.nunes@isel.pt (N.N.); filomena.martins@ciencias.ulisboa.pt (F.M.)

Table S1. Wavenumbers for each probe in methanol/formamide at 298.15 K (values in kK, 1 kK = 10^3 cm^{-1}).

Methanol/Formamide					
$x_{methanol}$	Betaine (30)	4-Nitroaniline	4-Nitrophenol	4-Nitroanisole	*N,N*-dimethyl-4-nitroaniline
1.00	19.386 ±0.023	27.010 ±0.000	32.101 ±0.041	32.701 ±0.023	25.641 ±0.008
0.90	19.543 ±0.009	26.842 ±0.014		32.493 ±0.028	25.426 ±0.007
0.80	19.631 ±0.000	26.692 ±0.023		32.410 ±0.020	25.219 ±0.007
0.70	19.674 ±0.012	26.483 ±0.000		32.180 ±0.026	25.063 ±0.000
0.60	19.706 ±0.009	26.455 ±0.000		32.051 ±0.017	25.063 ±0.000
0.50	19.701 ±0.006	26.256 ±0.008		31.923 ±0.020	24.647 ±0.007
0.40	19.716 ±0.000	26.224 ±0.008		31.832 ±0.019	24.554 ±0.007
0.30	19.695 ±0.004	26.114 ±0.008		31.719 ±0.012	24.522 ±0.000
0.20	19.698 ±0.004	26.082 ±0.011		31.666 ±0.016	24.288 ±0.007
0.10	19.645 ±0.011	26.006 ±0.015		31.539 ±0.011	24.192 ±0.019
0.00	19.614 ±0.007	25.950 ±0.013		31.420 ±0.011	24.093 ±0.007

Table S2. Wavenumbers for each probe in formamide/acetonitrile at 298.15 K (values in kK, 1 kK = 10^3 cm^{-1}).

Formamide/Acetonitrile					
$x_{acetonitrile}$	Betaine (30)	4-Nitroaniline	4-Nitrophenol	4-Nitroanisole	*N,N*-dimethyl-4-nitroaniline
1.00	16.013 ±0.000	27.457 ±0.000	32.552 ±0.000	32.418 ±0.012	25.407 ±0.000
0.90	18.129 ±0.019	27.304 ±0.014	31.933 ±0.022	32.432 ±0.012	25.339 ±0.012
0.80	18.472 ±0.014	27.037 ±0.008	31.762 ±0.026	32.244 ±0.012	25.142 ±0.007
0.70	18.710 ±0.004	26.915 ±0.008	31.674 ±0.045	32.170 ±0.012	25.063 ±0.000
0.60	18.834 ±0.007	26.714 ±0.008	31.581 ±0.019	32.092 ±0.000	25.063 ±0.000
0.50	18.981 ±0.008	26.539 ±0.014	31.577 ±0.032	31.928 ±0.000	24.712 ±0.007
0.40	19.133 ±0.004	26.418 ±0.007	31.458 ±0.032	31.969 ±0.033	24.785 ±0.007
0.30	19.233 ±0.007	26.302 ±0.011	31.296 ±0.011	31.759 ±0.012	24.466 ±0.007
0.20	19.390 ±0.004	26.183 ±0.008	31.111 ±0.057	31.686 ±0.000	24.331 ±0.000
0.10	19.423 ±0.007	26.110 ±0.000	30.834 ±0.044	31.632 ±0.012	24.221 ±0.007
0.00	19.614 ±0.007	25.950 ±0.013		31.420 ±0.011	24.093 ±0.007

Table S3. Wavenumbers for each probe in methanol/acetonitrile at 298.15 K. Data was taken from our previous work [3,23,24] (values in kK, 1 kK = 10^3 cm^{-1}).

	Methanol/Acetonitrile				
$x_{methanol}$	Betaine (30)	4-Nitroaniline	4-Nitrophenol	4-Nitroanisole	*N,N*-dimethyl-4-nitroaniline
1.000	19.388 ±0.021	27.012 ±0.000	32.101 ±0.040	32.701 ±0.023	25.641 ±0.008
0.955	19.443 ±0.022	27.056 ±0.012	32.088 ±0.034	32.775 ±0.071	25.632 ±0.008
0.910	19.417 ±0.000	27.086 ±0.000	32.072 ±0.000	32.683 ±0.029	25.602 ±0.000
0.875	19.417 ±0.000	27.108 ±0.062	32.092 ±0.000	32.623 ±0.026	25.523 ±0.000
0.810	19.443 ±0.022	27.091 ±0.020	32.117 ±0.082	32.545 ±0.012	25.500 ±0.027
0.750	19.455 ±0.000	27.076 ±0.008	32.058 ±0.012	32.549 ±0.084	25.476 ±0.007
0.725	19.455 ±0.000	27.179 ±0.044	32.065 ±0.012	32.531 ±0.000	25.461 ±0.045
0.695	19.417 ±0.000	27.164 ±0.009	32.051 ±0.000	32.514 ±0.084	25.441 ±0.007
0.625	19.455 ±0.000	27.115 ±0.000	32.099 ±0.012	32.489 ±0.023	25.407 ±0.011
0.595	19.417 ±0.000	27.205 ±0.048	32.082 ±0.021	32.473 ±0.020	25.423 ±0.012
0.500	19.380 ±0.000	27.159 ±0.000	32.044 ±0.012	32.432 ±0.012	25.394 ±0.000
0.425	19.279 ±0.010	27.218 ±0.000	32.065 ±0.012	32.493 ±0.035	25.385 ±0.015
0.375	19.212 ±0.000	27.268 ±0.009	32.065 ±0.012	32.446 ±0.000	25.389 ±0.007
0.333	19.182 ±0.021	27.284 ±0.008	32.106 ±0.012	32.475 ±0.012	25.407 ±0.000
0.250	19.022 ±0.016	27.307 ±0.000	32.141 ±0.012	32.468 ±0.000	25.411 ±0.007
0.190	18.815 ±0.000	27.307 ±0.000	32.203 ±0.012	32.534 ±0.103	25.394 ±0.000
0.143	18.657 ±0.000	27.405 ±0.000	32.210 ±0.012	32.452 ±0.011	25.394 ±0.022
0.081	18.193 ±0.019	27.427 ±0.000	32.293 ±0.012	32.498 ±0.071	25.402 ±0.007
0.045	17.841 ±0.000	27.447 ±0.009	32.439 ±0.012	32.425 ±0.000	25.419 ±0.000
0.000	16.013 ±0.000	27.457 ±0.000	32.552 ±0.000	32.418 ±0.012	25.407 ±0.000

Table S4. Wavenumbers for each probe in the ternary mixture methanol/formamide/acetonitrile at 298.15 K (values in kK, 1 kK = 10^3 cm^{-1}).

		Methanol/Formamide/Acetonitrile				
$x_{methanol}$	$x_{formamide}$	Betaine(30)	4-Nitroaniline	4-Nitrophenol	4-Nitroanisole	*N,N*-dimethyl-4-nitroaniline
0.333	0.333	19.411 ±0.016	26.667 ±0.009	31.012 ±0.031	32.113 ±0.000	24.892 ±0.017
0.500	0.250	19.520 ±0.023	26.686 ±0.007		32.154 ±0.000	25.031 ±0.024
0.500	0.150	19.463 ±0.000	26.865 ±0.022		32.489 ±0.000	25.190 ±0.025
0.500	0.050	19.406 ±0.020	27.064 ±0.013	32.129 ±0.020	32.489 ±0.000	25.305 ±0.016
0.500	0.350	19.576 ±0.012	26.488 ±0.007		32.072 ±0.000	24.884 ±0.009
0.250	0.500	19.430 ±0.015	26.377 ±0.029		31.888 ±0.000	24.656 ±0.015
0.350	0.500	19.552 ±0.018	26.316 ±0.000		31.926 ±0.032	24.677 ±0.021
0.150	0.500	19.323 ±0.015	26.511 ±0.000		31.939 ±0.011	24.685 ±0.030
0.050	0.500	19.125 ±0.020	26.499 ±0.027	31.476 ±0.015	31.919 ±0.011	24.717 ±0.027
0.250	0.250	19.320 ±0.011	26.856 ±0.031	31.632 ±0.016	32.154 ±0.000	25.031 ±0.023
0.350	0.150	19.357 ±0.013	26.945 ±0.039		32.489 ±0.000	25.178 ±0.016
0.450	0.050	19.330 ±0.014	27.073 ±0.018	31.915 ±0.011	32.489 ±0.000	25.308 ±0.007
0.150	0.350	19.137 ±0.007	26.748 ±0.015	31.586 ±0.000	32.134 ±0.000	24.896 ±0.010
0.750	0.125	19.538 ±0.021	26.852 ±0.032		32.489 ±0.000	25.292 ±0.011
0.125	0.750	19.481 ±0.017	26.128 ±0.014		31.612 ±0.020	24.366 ±0.027
0.125	0.125	18.811 ±0.000	27.102 ±0.014	31.888 ±0.000	32.489 ±0.000	25.238 ±0.022
0.050	0.050	18.208 ±0.000	27.281 ±0.010	32.092 ±0.000	32.489 ±0.000	25.305 ±0.012
0.050	0.900	19.516 ±0.023	25.988 ±0.000		31.520 ±0.030	24.199 ±0.025
0.900	0.050	19.481 ±0.005	26.899 ±0.029		32.531 ±0.000	25.465 ±0.024
0.950	0.025	19.433 ±0.021	26.940 ±0.006		32.680 ±0.000	25.569 ±0.007
0.025	0.950	19.496 ±0.014	25.961 ±0.000		31.449 ±0.023	24.136 ±0.015
0.025	0.025	17.745 ±0.016	27.349 ±0.012	32.113 ±0.000	32.489 ±0.000	25.376 ±0.006

Table S5. Adjusted parameters for the Bosch and Rosés model for betaine (30) at 298.15 K. Data referring to methanol/acetonitrile was taken from our previous work [23,24] .

	Mixture		
Parameter	Methanol/Formamide	Formamide/Acetonitrile	Methanol/Acetonitrile
Y_1	19.386	19.46	19.39
s	±0.05	±0.1	±0.02
CL (%)	100	100	100
Y_2	19.612	16.00	16.02
s	±0.006	±0.05	±0.03
CL (%)	100	100	100
Y_{12}	19.83	**19.09**	19.53
s	±0.03	**±0.06**	±0.02
CL (%)	100	---	100
$f_{2/1}$	2.7	0.12	0.4
s	±0.5	±0.02	±0.2
CL (%)	99.7	100	88.0
$f_{12/1}$	4.9	**1.12**	7.1
s	±0.7	**±0.02**	±4.4
CL (%)	99.9	---	86.9
m	2	1	2
r^2	0.998	0.993	0.999
s_{adj}	0.005	0.089	0.023
F	559	465	4239
N	10	10	19

Table S6. Adjusted parameters for the Bosch and Rosés model for 4-nitroaniline at 298.15 K. Data referring to methanol/acetonitrile was taken from our previous work [3].

	Mixture		
Parameter	Methanol/Formamide	Formamide/Acetonitrile	Methanol/Acetonitrile
Y_1	27.02	25.97	27.05
s	±0.03	±0.02	±0.02
CL (%)	100	100	100
Y_2	25.95	27.48	27.47
s	±0.02	±0.02	±0.02
CL (%)	100	100	100
Y_{12}	**26.30**	**26.57**	**27.20**
s	**±0.03**	**±0.03**	**±0.02**
CL (%)	---	---	---
$f_{2/1}$	2.0	0.65	0.6
s	±0.2	±0.05	±0.1
CL (%)	100	100	100
$f_{12/1}$	**3.0**	**1.65**	**1.6**
s	**±0.2**	**±0.05**	**±0.1**
CL (%)	---	---	---
m	1	1	1
r^2	0.993	0.997	0.955
s_{adj}	0.029	0.026	0.029
F	580	1408	180
N	11	11	20

Table S7. Adjusted parameters for the Bosch and Rosés model for 4-nitrophenol at 298.15 K. Data referring to methanol/acetonitrile was taken from our previous work [3] .

	Mixture		
Parameter	Methanol/Formamide	Formamide/Acetonitrile	Methanol/Acetonitrile
Y_1			32.10
s	---	---	±0.01
CL (%)			100
Y_2			32.56
s	---	---	±0.02
CL (%)			100
Y_{12}			31.95
s	---	---	±0.06
CL (%)			100
$f_{2/1}$			0.11
s	---	---	±0.08
CL (%)			80.6
$f_{12/1}$			0.7
s	---	---	±0.7
CL (%)			69.3
m	---	---	2
r^2			0.977
s_{adj}			0.020
F	---	---	160
N			20

Table S8. Adjusted parameters for the Bosch and Rosés model for 4-nitroanisole at 298.15 K. Data referring to methanol/acetonitrile was taken from our previous work [3].

	Mixture		
Parameter	Methanol/Formamide	Formamide/Acetonitrile	Methanol/Acetonitrile
Y_1	32.70	31.46	32.71
s	±0.03	±0.04	±0.02
CL (%)	100	100	100
Y_2	31.45	32.44	32.43
s	±0.02	±0.04	±0.01
CL (%)	100	100	100
Y_{12}	**31.94**	**31.99**	**32.48**
s	**±0.03**	**±0.05**	**±0.01**
CL (%)			
$f_{2/1}$	1.5	1.2	4.8
s	±0.1	±0.2	±1.1
CL (%)	100	99.9	99.9
$f_{12/1}$	**2.5**	**2.2**	**5.8**
s	**±0.1**	**±0.2**	**±1.1**
CL (%)			
m	1	1	1
r^2	0.996	0.979	0.931
s_{adj}	0.028	0.048	0.020
F	898	187	88
N	11	11	16

Table S9. Adjusted parameters for the Bosch and Rosés model for *N,N*-dimethyl-4-nitroaniline at 298.15 K. Data referring to methanol/acetonitrile was taken from our previous work [3].

	Mixture		
Parameter	Methanol/Formamide	Formamide/Acetonitrile	Methanol/Acetonitrile
Y_1	25.65	24.06	25.65
s	±0.04	±0.06	±0.01
CL (%)	100	100	100
Y_2	24.09	25.42	25.41
s	±0.03	±0.06	±0.01
CL (%)	100	100	100
Y_{12}	**24.71**	**24.81**	23.40
s	**±0.04**	**±0.08**	±9.6
CL (%)			97.1
$f_{2/1}$	1.5	1.2	8.7
s	±0.2	±0.2	±3.0
CL (%)	100	99.9	98.9
$f_{12/1}$	**2.5**	**2.2**	0.2
s	**±0.2**	**±0.2**	±0.8
CL (%)			16.2
m	1	1	2
r^2	0.995	0.977	0.989
s_{adj}	0.038	0.069	0.009
F	691	170	320
N	10	11	19

Table S10. Adjusted parameters for the Bosch and Rosés model for each probe in the ternary mixture methanol/formamide/acetonitrile at 298.15 K.

Parameter	Betaine (30)	4-Nitroaniline	4-Nitrophenol	4-Nitroanisole	*N,N*-dimethyl-4-nitroaniline
Y_1	19.40	26.99		32.69	25.635
s	±0.03	±0.01	---	±0.03	±0.008
CL (%)	100	100		100	100
Y_2	19.50	25.95		31.43	24.093
s	±0.02	±0.02	---	±0.03	±0.009
CL (%)	100	100		100	100
Y_3	16.03	27.43		32.45	25.425
s	±0.04	±0.02	---	±0.02	±0.007
CL (%)	100	100		100	100
Y_{12}	19.9				
s	±0.1	---	---	---	---
CL (%)	100				
Y_{13}	19.54				25.19
s	±0.03	---	---	---	±0.04
CL (%)	100				100
$f_{2/1}$	1.6	1.8		1.6	1.48
s	±0.8	±0.1	---	±0.2	±0.06
CL (%)	95.4	100		100	100
$f_{3/1}$	0.2	1.3		1.9	1.48
s	±0.1	±0.1	---	±0.3	±0.06
CL (%)	94.7	100		100	100
$f_{12/1}$	3.9				
s	±2.7	---	---	---	---
CL (%)	83.7				
$f_{13/1}$	4.1				1.4
s	±2.1	---	---	---	±0.2
CL (%)	93.8				100
$f_{123/1}$	2.5				
s	±1.4	---	---	---	---
CL (%)	92.5				
Y_{123}	19.0				
s	±0.4	---	---	---	---
CL (%)	100				
m	2	1	---	1	2
r^2	0.995	0.992		0.974	0.999
s_{adj}	0.040	0.039	---	0.058	0.016
F	849	1660		530	6766
N	56	61		61	53

Table S11. Kamlet-Taft parameters for the methanol/formamide mixture at 298.15 K.

Methanol/Formamide						
$x_{methanol}$	π^*_{OMe}	π^*_{NMe2}	α_{OMe}	α_{NMe2}	β_{OH}	β_{NH2}
1.0	0.591	0.721	0.970	1.002	0.725	0.621
0.9	0.678	0.782	0.933	0.979		0.605
0.8	0.713	0.841	0.922	0.945		0.585
0.7	0.808	0.886	0.860	0.915		0.606
0.6	0.862	0.886	0.827	0.921		0.616
0.5	0.915	1.004	0.787	0.820		0.540
0.4	0.953	1.030	0.762	0.800		0.518
0.3	1.000	1.039	0.725	0.789		0.546
0.2	1.023	1.106	0.710	0.733		0.474
0.1	1.075	1.133	0.663	0.700		0.468
0.0	1.125	1.161	0.622	0.670		0.453

Table S12. Kamlet-Taft parameters for the formamide/acetonitrile mixture at 298.15 K.

Formamide/Acetonitrile						
$x_{acetonitrile}$	π^*_{OMe}	π^*_{NMe2}	α_{OMe}	α_{NMe2}	β_{OH}	β_{NH2}
1.0	0.709	0.788	0.345	0.329	0.352	0.375
0.9	0.703	0.807	0.688	0.699	0.669	0.407
0.8	0.782	0.863	0.687	0.715	0.656	0.433
0.7	0.813	0.886	0.703	0.739	0.661	0.449
0.6	0.845	0.886	0.699	0.762	0.667	0.522
0.5	0.913	0.985	0.674	0.704	0.584	0.460
0.4	0.896	0.964	0.710	0.749	0.664	0.530
0.3	0.984	1.055	0.663	0.691	0.636	0.458
0.2	1.014	1.093	0.666	0.687	0.690	0.454
0.1	1.037	1.125	0.655	0.667	0.801	0.441
0.0	1.125	1.161	0.622	0.670		0.453

Table S13. Kamlet-Taft parameters for the methanol/acetonitrile mixture at 298.15 K. Data for this mixture was taken from our previous work [3].

Methanol/Acetonitrile						
$x_{methanol}$	π^*_{OMe}	π^*_{NMe2}	α_{OMe}	α_{NMe2}	β_{OH}	β_{NH2}
1.000	0.591	0.721	0.970	1.002	0.725	0.620
0.955	0.561	0.724	1.002	1.006	0.769	0.601
0.910	0.599	0.732	0.970	0.984	0.730	0.580
0.875	0.624	0.755	0.952	0.966	0.688	0.544
0.810	0.656	0.761	0.933	0.956	0.635	0.541
0.750	0.655	0.768	0.936	0.955	0.667	0.538
0.725	0.662	0.772	0.930	0.951	0.654	0.496
0.695	0.669	0.778	0.919	0.939	0.652	0.494
0.625	0.680	0.788	0.918	0.938	0.615	0.499
0.595	0.686	0.783	0.907	0.931	0.615	0.472
0.500	0.703	0.792	0.889	0.915	0.613	0.479
0.425	0.678	0.794	0.891	0.906	0.634	0.454
0.375	0.697	0.793	0.866	0.888	0.610	0.438
0.333	0.686	0.788	0.870	0.889	0.604	0.438
0.250	0.689	0.787	0.842	0.861	0.583	0.431
0.190	0.661	0.792	0.829	0.833	0.587	0.425
0.143	0.695	0.792	0.779	0.794	0.540	0.390
0.081	0.676	0.789	0.718	0.722	0.523	0.385
0.045	0.706	0.784	0.640	0.653	0.412	0.383
0.000	0.709	0.788	0.345	0.337	0.352	0.375

Table S14. Kamlet-Taft parameters for the methanol/formamide/acetonitrile mixture at 298.15 K.

Methanol/Formamide/Acetonitrile							
$x_{methanol}$	$x_{formamide}$	π^*_{OMe}	π^*_{NMe2}	α_{OMe}	α_{NMe2}	β_{OH}	β_{NH2}
0.333	0.333	0.836	0.885	0.798	0.826	0.960	0.478
0.500	0.250	0.819	0.857	0.828	0.880		0.521
0.500	0.150	0.680	0.765	0.919	0.907		0.513
0.500	0.050	0.680	0.748	0.910	0.925	0.600	0.481
0.500	0.350	0.853	0.895	0.812	0.854		0.540
0.250	0.500	0.930	0.966	0.733	0.773		0.499
0.350	0.500	0.914	0.955	0.764	0.800		0.529
0.150	0.500	0.909	0.951	0.731	0.760		0.461
0.050	0.500	0.917	0.950	0.694	0.732	0.629	0.477
0.250	0.250	0.819	0.857	0.796	0.843	0.674	0.459
0.350	0.150	0.680	0.766	0.902	0.885		0.479
0.450	0.050	0.680	0.748	0.898	0.912	0.707	0.480
0.150	0.350	0.828	0.880	0.760	0.777	0.686	0.450
0.750	0.125	0.680	0.750	0.931	0.946		0.554
0.125	0.750	1.045	1.064	0.659	0.712		0.486
0.125	0.125	0.680	0.758	0.814	0.800	0.720	0.444
0.050	0.050	0.680	0.748	0.718	0.706	0.618	0.403
0.050	0.900	1.083	1.107	0.637	0.678		0.477
0.900	0.050	0.662	0.717	0.934	0.977		0.598
0.950	0.025	0.600	0.671	0.971	0.993		0.621
0.025	0.950	1.113	1.131	0.612	0.659		0.464
0.025	0.025	0.680	0.738	0.644	0.638	0.608	0.404

Table S15. Wavenumbers for the second band of 4-nitrophenol in the binary mixture methanol/formamide at 298.15 K (values in kK, 1 kK = 10^3 cm^{-1}).

Methanol/Formamide	
$x_{methanol}$	4-Nitrophenol
1.00	
0.90	25.528 ±0.008
0.80	25.332 ±0.012
0.70	25.134 ±0.007
0.60	25.063 ±0.000
0.50	25.063 ±0.000
0.40	24.694 ±0.012
0.30	24.394 ±0.007
0.20	24.399 ±0.011
0.10	24.213 ±0.000
0.00	24.038 ±0.000

Table S16. Wavenumbers for the second band of 4-nitrophenol in the binary mixture formamide/acetonitrile at 298.15 K (values in kK, 1 kK = 10^3 cm^{-1}).

Formamide/Acetonitrile	
$x_{acetonitrile}$	4-Nitrophenol
1.00	
0.90	
0.80	
0.70	24.260 ±0.012
0.60	24.219 ±0.012
0.50	24.244 ±0.014
0.40	24.208 ±0.006
0.30	24.166 ±0.000
0.20	24.147 ±0.007
0.10	24.103 ±0.016
0.00	24.038 ±0.000

Table S17. Wavenumbers for the second band of 4-nitrophenol in the ternary mixture methanol/formamide/ acetonitrile at 298.15 K (values in kK, 1 kK = 10^3 cm^{-1}).

Methanol/Formamide/Acetonitrile		
$x_{methanol}$	$x_{formamide}$	4-Nitrophenol
0.333	0.333	24.773 ±0.020
0.500	0.250	24.975 ±0.022
0.500	0.150	25.042 ±0.031
0.500	0.050	25.439 ±0.031
0.500	0.350	24.874 ±0.004
0.250	0.500	24.561 ±0.019
0.350	0.500	24.673 ±0.020
0.150	0.500	24.456 ±0.021
0.050	0.500	24.285 ±0.004
0.250	0.250	24.728 ±0.000
0.350	0.150	24.999 ±0.024
0.450	0.050	25.445 ±0.000
0.150	0.350	24.594 ±0.000
0.750	0.125	25.305 ±0.011
0.125	0.750	24.315 ±0.014
0.125	0.125	
0.050	0.050	
0.050	0.900	24.136 ±0.020
0.900	0.050	25.512 ±0.005
0.950	0.025	25.570 ±0.007
0.025	0.950	24.150 ±0.023
0.025	0.025	

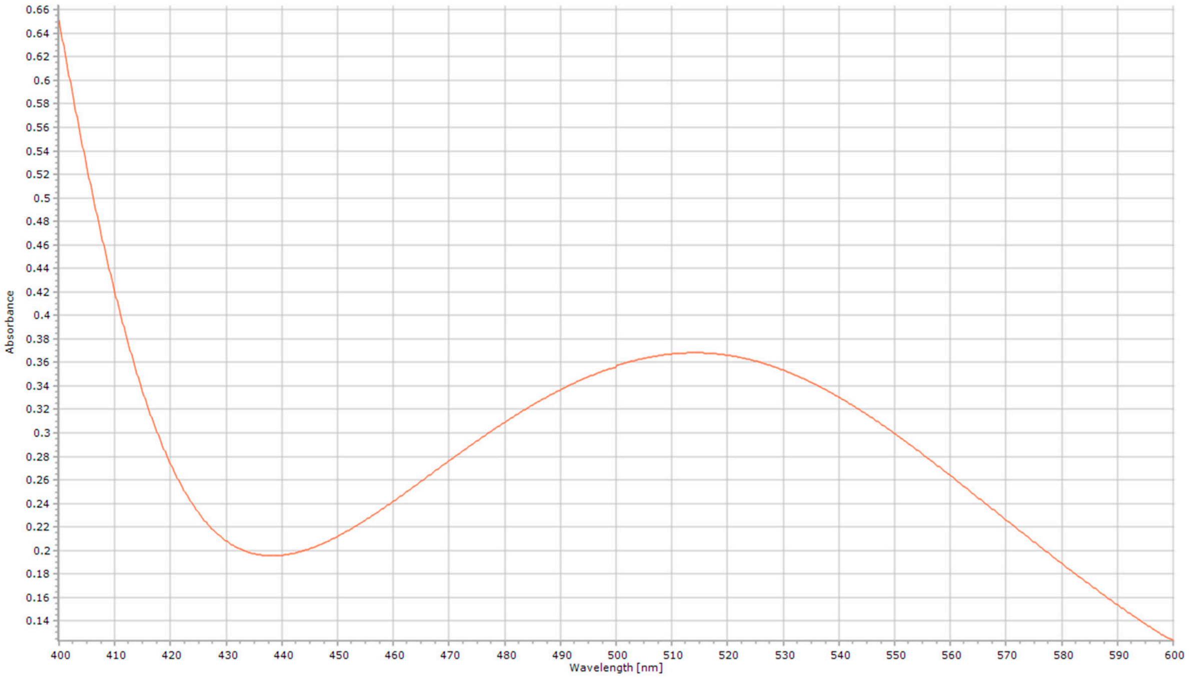

Figure S1. Spectrum of Betaine (30) in the ternary mixture methanol/formamide/acetonitrile (0.333/0.333/0.333).
